# Colonic stenting in the management of malignant intestinal obstruction in elderly patients

**DOI:** 10.1186/1471-2482-13-S1-A27

**Published:** 2013-09-16

**Authors:** A Maffioli, A Bondurri, G Manes, D Cavallo, S Callioni, PG Danelli

**Affiliations:** 1 Department of General Surgery 1, Department of Gastroenterology – “Luigi Sacco” Hospital / University of Milan, Milan, Italy

## Introduction

Acute intestinal obstruction occurs in 7-30% of patients with colorectal carcinoma, especially if tumour is located at or distal to the splenic flexure. Traditionally, emergency surgical decompression is the treatment option, with the resection of obstructing tumour and often with the creation of defunctioning stoma. Emergency surgical decompression is very effective, but it is associated to high mortality rate, depending on patients comorbidities, anaesthetic and emergency surgery risks. Moreover stoma causes high morbidity rate and a worsening in quality of life, with up to 60% patients that would never be reversed [1].

An alternative to surgery is intraluminal colorectal stenting, first described by Dohmoto in 1991 in 19 patients with non-resectable metastatic rectal cancer. In 1993 Tejero used metallic stents in patients with colon obstruction as “bridge to surgery” [2].

Since its introduction self-expandable metal stent (SEMS) has increasingly been used for malignant colon obstruction and several studies showed its efficacy in relieving the obstruction offering good palliation, and, whenever possible, avoiding emergency surgery and facilitating single-stage surgery, reducing stoma creation.

Colonic stenting is also associated to complications, as intestinal perforation, stent migration and clinical failure.

Nowadays there is debate concerning five-years survival and cancer-specific mortality: it seems that five-years survival is lower and cancer-specific mortality is higher in SEMS patients compared to urgent surgery patients, because of delayed surgery [3].

The aim of the study was to review our experience and assess the effectiveness of colonic stenting in malignant colon obstruction, as a “bridge to surgery” or as a palliative treatment, in terms of safety, efficacy and clinical outcomes.

## Methods

During the studied period (2008-2013), 18 patients over 80 were selected to undergo endoscopic stenting for malignant colon obstruction in L. Sacco University Hospital, Milan.

The main indication for stenting was obstructive colon cancer, diagnosed by clinical evaluation, abdomen x-rays and CT-scan. Exclusion criteria were perforation, peritonitis or intestinal bleeding.

According to the characteristics of the stricture stents with different length (140, 110, 100 mm) and diameter (22, 24 mm) were placed. In case of palliative treatment, covered stents were preferred. All the stents were placed under endoscopic and fluoroscopic guide.

After 24 hours from stent insertion, an abdominal x-ray was obtained routinely.

Technical success of the procedure was defined as the right placement of the stent across the stricture; clinical success was defined as colonic decompression and relief of obstructive symptoms within 96 hours from the procedure.

In the group “bridge to surgery”, the interval between stent placement and elective surgery was decided according to clinical conditions and bowel function of the patient.

## Results

These are preliminary results of a study conducted on a total of 18 patients (mean age 83,4 years, range 80-100, 12 men, 7 women). The procedure was performed as a bridge to surgery in 8 patients (44,5%, mean age 80,6) and for palliative management in 10 patients (55,5%, mean age 85,0).

The site of obstruction was in transverse colon in 1 patient (5,6%), left colon in 3 patients (16,7%), rectosigmoid in 14 patients (77,8%).

Technical success was obtained in 15 patients (83,3%); failure occurred in 3 patients (16,7%), 2 in the “bridge to surgery” group and 1 in palliation group. Failure was due to perforation during the procedure (n=1), a narrowing stricture (n=1) and incomplete stent expansion (n=1).

Clinical success was obtained in all the 15 patients with successful stent placement, within the first day after the procedure, except in one case in which occurred after 4 days.

Early complications (within the first week after stent placement) occurred in 4 patients (22,2%), 3 in the “bridge to surgery group” and 1 in the palliation group. Early complications were 2 cases of intestinal bleeding and 1 case of micro-perforation, conservatively treated, and 1 case of rectal perforation that required emergency surgery. There were no cases of stent obstruction, migration or rectal tenesmus.

After the first week, no complications were recorded.

In the group of “bridge to surgery” 6 patients underwent elective surgery (75%) without the need of stoma creation. Median time between stent placement and elective surgery was 8,7 days (range 3-13).

In the case of rectal perforation, a subtotal colectomy with diverting ileostomy was performed; the patient with narrowing stricture underwent emergency Hartmann procedure.

## Conclusions

Colonic endoscopic stenting is safe and effective in the management of malignant colorectal obstruction for palliative care or “bridge to a surgery”, with a low complication rate (22,2%).

In the “bridge to surgery” group, stent placement is an option to improve clinical conditions and decrease bowel dilatation before elective surgery. These make colonic stenting a good alternative treatment to emergency colostomy or Hartmann procedure.

Moreover it provides a good long-term palliation, without the need of surgical procedure and stoma creation, with an improvement in the quality of life.

Our promising preliminary results about colonic stenting in elderly patients should be confirmed by a prospective randomized trial on a higher number of patients.
